# Determination of Water-Soluble Components of Abdominal Secretion of Grasshopper (*Chorthippus* spp.) by GC/MS/MS in Search for Potential Wound Healing Agents

**DOI:** 10.1007/s10337-014-2679-8

**Published:** 2014-04-29

**Authors:** Magdalena Buszewska-Forajta, Wiktoria Struck-Lewicka, Renata Bujak, Danuta Siluk, Roman Kaliszan

**Affiliations:** Department of Biopharmaceutics and Pharmacodynamics, Medical University of Gdańsk, Al. Gen. J. Hallera 107, 80-416 Gdańsk, Poland

**Keywords:** GC/MS/MS, *Chorthippus* spp., Grasshoppers’ abdominal secretion, Metabolomics, Water-soluble compounds, Wound healing

## Abstract

Wound healing is still a serious medical problem due to process complexity and lack of effective medicaments. This is particularly true in the treatment of wounds arising in the course of such diseases as AIDS or diabetes. Therefore, scientific efforts are focused on the search for new compounds of natural origin, which could be used as medicines or evaluated for subsequent drug design. In folk medicine, grasshopper (*Chorthippus* spp.) abdominal secretion has been used to accelerate the wound healing process. In this context, the knowledge of the composition of grasshopper abdominal secretion is crucial. The aim of this study was to determine the main water-soluble components of grasshopper abdominal secretion with the use of GC/MS/MS. Liquid–liquid extraction was used as a pretreatment method to clean up, concentrate and fractionate compounds from the complex insect matrix. To obtain more stable and volatile compounds, necessary for GC/MS/MS analysis, a double-step derivatization process was carried out with the use of methoxyamine hydrochloride and a mixture of bis-*N,O*-trimethylsilyl trifluoroacetamide and chlorotrimethylsilane. As a result, 2,108 compounds were identified, mainly as amino acids, carbohydrates and organic acids. Some of the identified compounds are emphasized due to antimicrobial, antifungal or antioxidant activities reported in the literature. Moreover, a set of compounds characteristic for *Chorthippus* spp. samples has been selected. In the last part of the study, a statistical analysis was performed to demonstrate differences in composition of aqueous fractions of abdominal secretions from grasshoppers collected at two distant locations: Starogard Gdański and Łubianka meadows.

## Introduction

Metabolomics is a relatively new discipline of bioscience aiming at understanding of the metabolic state of a biological system. Results of qualitative and quantitative determinations of metabolites in the organism can be related to differences in biological reactions to various stimuli [1]. Genetic and environmental factors can affect metabolites concentration, which in turn, can determine several physiological responses [2].

Metabolomics, as a part of functional genomics, plays an important role in medically oriented research on insects and plants [3]. Fingerprint analysis, performed by complementary gas and liquid chromatography, coupled with the mass spectrometry technique, is a fundamental approach in metabolomic studies. One of mass spectrometry’s beneficial features is its usefulness in identification of metabolites in complex biological material. Such identified metabolites might next be recognized as specific biomarkers or so-called “lead” structures for rational drug design.

Substances with biological activity have been sought since ages but still searching for new bioactive agents is necessary. Nowadays, much attention is dedicated to insect’s metabolites, because of their unique, myotropic and cardiotropic properties [4]. The usage of bioactive compounds obtained from herbs, insects or animals has been highlighted in folk medicine [5]. According to the ethnographic literature [5] and childhood’s memories of one of the authors (Roman Kaliszan), villagers of West-Central and South Poland used the grasshopper abdominal secretion for wound healing and in wart treatment. Interestingly, the grasshopper abdominal secretion is characterized by extraordinary rheological properties.

The most common Polish species of grasshopper genus are *Chorthippus biguttulus, Chorthippus montanus* and *Chorthippus parallelus.* The common territories of occurrence of the three species are sunny meadows and fields.

On the basis of folk premises, an ointment-like material, squeezed out from grasshopper ovipositor, facilitates healing of wounds and scars. Ovipositor is an egg-laying structure at the end of the abdomen in many female insects. One of the most important life processes of female grasshoppers is reproduction and laying eggs into the solid surface, which are highly dependent on insect’s hormone secretion [4, 6–8]. The eggs are suspended in abdominal secretion produced by gonads [4]. A characteristic feature of secretion is its protective role. It should protect the insects’ eggs against pathogens, dehydration, and fill the space between eggs [7]. It is known also as a thermo-isolating and nourishing material for eggs [8].

The main aim of this study was to determine the metabolomic profile of water-soluble compounds, contained in grasshoppers’ abdominal secretion, with the use of gas chromatography coupled with mass spectrometry (GC/MS).

A wide range of analytical platforms are available to perform metabolomic analysis. In the group of analytical methods used in metabolomics study NMR, liquid and gas chromatography coupled with mass spectrometry, capillary electrophoresis, infrared and Raman spectroscopies could be found [9]. Using GC/MS is profitable due to the possibility of determining a wide range of chemical structures, such as amino acids, carbohydrates, sugars, fatty acids and lipids, in complex matrices. This platform is dedicated to determine volatile compounds as well as those, which could easily be derivatized [10]. Moreover, it is the best one to perform analysis of polar compounds, like amino acids. The advantages of GC/MS are: its high sensitivity, reproducibility and possibility to determine large amounts of compounds (200–300) in a single analysis. A performed advantage of GC/MS is a possibility of compounds’ identification. It is done by comparison of mass spectra and retention time obtained for each compound with information accumulated in the reference library, like NIST. It makes compounds’ identification straightforward and quicker than in other platforms (CZE/MS, LC/MS). Showed features allow scientists to recognize the GC/MS technique as versatile as well as conveniently applicable in biomedical research [11].

Below we report identification of water-soluble compounds profile of grasshopper abdominal secretion, determined with the use of gas chromatography coupled with mass spectrometry. To our best knowledge, it is the first report on the composition of water-soluble compounds in grasshopper abdominal secretion.

## Materials and Methods

### Chemicals

The derivatizing agents, methoxyamine hydrochloride, pyridine, bis-*N,O-*trimethylsilyl trifluoroacetamide (BSTFA) and chlorotrimethylsilane (TMCS), were purchased from Sigma-Aldrich, St. Louis, MO, USA. Hexane and methanol were purchased from J.T. Baker, Deventer, Netherlands, and chloroform from POCH, Gliwice, Poland. Deionized water was obtained with Milli-RO and Milli-QPlus instrumentation from Millipore, Billerica, MA, USA.

### Insects Collection

Female grasshopper insects *Chorthippus* spp. were collected in Łubianka and Starogard Gdański meadows. Collection conditions (day and area of collection, weather) were documented. Each insect was macroscopically and microscopically recognized with the use of stereoscopic microscope (MST 132 LAB TK PZO, Warsaw, Poland). Moreover, anatomical differences in ovipositor and wings (the ratio of length to width) between males and females were useful in gender identification. Immediately, insects were dropped into liquid nitrogen to deactivate proteolytic enzymes. Each insect was put into cryo-container and stored at −80 °C until analysis. In the day of analysis, the insects were thawed and secretion was obtained from each specimen. Samples were divided into two groups, due to the insect collection area. Each group was composed of an equal number of 20 samples.

### Sample Extraction Procedure

Grasshoppers’ abdominal secretion was extracted with Bligh and Dyer method [12]. The procedure was normalized to the mass of grasshopper abdominal secretion. Firstly, water was added to secretion in a strictly determined volume: for 20 mg of biomaterial from each grasshopper, 80 µL of water was added. Then, 300 µL of a mixture chloroform:methanol (1:2, *v/v*) and 100 µL of pure chloroform were added. The last step of extraction procedure was the addition of 100 µL of water.

As a result of extraction, two fractions were obtained: organic and aqueous ones. The aqueous fraction, in a volume of 50 µL, was transferred to glass vials. Then, the solution was evaporated to dryness with the use of vacuum concentrator (Genevac Inc., Valley Cottage, NY, USA) within 3 min at 30 °C. Dry residues underwent derivatization procedure.

The results of analysis of the organic fraction have previously been reported by us elsewhere [13].

### Quality Control Samples Preparation

Quality control (QC) samples were prepared by mixing in a glass vial 20 μL of each aqueous fraction obtained from 40 samples. Then, 50 µL of the mixture was processed using the same pretreatment procedure as for regular samples.

Blank samples, containing water (100 µL), were prepared using the same pretreatment procedure as in case of insect secretion samples.

### Derivatization

Derivatization process was carried out in two steps. Firstly, a 2-h oximation reaction was performed, in room temperature, with the use of 20 µL of methoxyamine hydrochloride in pyridine solution (15 mg mL^−1^). In the second step, each sample was silanized by the addition of 20 μL of a mixture of BSTFA/TMCS (99/1, *v/v*) for 60 min at 97 °C in a thermoblock. After derivatization process, 100 μL of hexane was added to each cooled down sample and vortex mixed for 2 min. 2 μL of derivatized sample was injected into the system with the split mode (split ratio 1:3). The samples were randomized with the use of Excel software (Excel, Microsoft Office 2007).

### Instrumentation

To perform the analysis, a Shimadzu GC–MS System (Kyoto, Japan) was used. The system was composed of an AOC-20S auto-sampler, AOC-20i autoinjector and a gas chromatograph GC 2010 plus, coupled with a triple quadrupole mass spectrometer. In post-analysis processing of the obtained data, Mass GC/MS Solution Software version 4.01 (Shimadzu, Kyoto, Japan) was used.

### GC/MS Analysis

The derivatized samples were separated on a Zebron ZB-5MS (30 m × 0.25 mm i.d., 0.25 μm film thickness) capillary CG column (Phenomenex, Torrance, CA, USA). Analyses were performed with the helium as a carrier gas at a constant pressure mode (65.2 kPa). The separation was carried out in a gradient temperature program. The oven temperature was maintained at 45 °C for 5 min, ramped to 70 °C at 3 °C min^−1^, increased at a rate of 10 °C min^−1^ to 220 °C, ramped to 250 °C with the grade 3 °C min^−1^ and held for 3 min, and then increased at a rate of 10 °C min^−1^ and held again for 15 min. The total run time was 63 min. The injector, ion source and interface temperature were set at 320 °C, 220 °C and 320 °C, respectively. The mass spectra of grasshoppers’ abdominal secretion in 10–1,000 *m/z* range were recorded by the positive electron impact ionization mass spectrometer, with ionization voltage of 70 eV.

### Data Preprocessing

The obtained chromatograms were processed with the use of the method created within the GC/MS Solution Software Postrun Analysis (Shimadzu, Kyoto, Japan), after its optimization. The data were processed with the following parameters of autoarea mode: maximum peak number = 500, width time = 2 s, a standard smoothing method, minimum peak area = 50,000. Next, each chemical compound was identified based on its retention time, retention index and mass spectrum included in NIST (National Institute of Standards and Technology) library to minimize or exclude the process errors, as well as to obtain the matrix with known compounds. As a result, we received a matrix composed of 2,108 compounds with match factor 80 % (called as variables) and 40 samples (objects).

The presented data set contained 2,108 compounds that were determined in all the analyzed samples (*n* = 40). Some of these compounds were present only in a single analyzed sample; some were just characterized as artifacts (recognized as reagents of derivatization process or solely its intermediate or final products). Other compounds had such low intensity that it was impossible to identify them using AMDIS software, therefore, sample recalibration process was impossible to succeed. To obtain the most informative data matrix with identified compounds without uninformative noise or artifacts, we decided to filtrate our data set, especially in a view point that data filtration is recommended for untargeted analysis in metabolomics [14]. The matrix was filtered with the use of Excel Software (Excel, Microsoft Office, 2007) and after filtration the new matrix limited to 69 compounds, was created. Data filtration procedure with assumption that compounds have to be present in one out of two groups (Starogard Gdański/Łubianka meadows) in at least 60 % of samples, enabled us to obtain a new set of 69 compounds, which are identified with high match score—of more than 80 %. More than 2,000 compounds were excluded due to either their absence under frequency or match factor condition applied. In addition, compounds with intensities at a noise level, were also excluded to avoid mistakes in identification.

Among these 69 compounds, 52 were present in the samples of both groups. 12 compounds were detected only in the samples collected from Łubianka meadows and 5 only in samples from Starogard Gdański. This matrix was also checked under drift retention time conditions (RT window = 0.1 min) and was used for subsequent statistical analysis. Moreover, another filtration procedure was applied, that selected compounds present in at least 80 % in one out of the two groups. The selected compounds, occurring at 80 % frequency level, are presented in Table 1.

**Table 1:** Compounds identified in all samples with at least 80 % frequency level

No.	Compound name	*t*_R_ (min)	Specific ions *m/z*	NIST match	Chemical group	MW	Frequency (%)
1.	1,4-Butanediamine, *N*,*N*,*N*′,*N*′-tetrakis(trimethylsilyl)-	26.84	214, 174, 148, 73	92	Amine	376	82.5
2.	2,4,4-Trimethyl-1-pentanol, trifluoroacetate	21.77	127, 97, 57	81	Alcohol	226	85
3.	2-Pyrrolidone-5-carboxylic acid, trimethylsilyl ester	24.30	186, 156, 84, 73, 56	81	Organic acid derivatives	201	87.5
4.	Alanine, phenyl- trimethylsilyl ester, dl-	24.54	222, 204, 148, 130, 120, 103, 91, 73, 45	92	Amino acid derivatives	237	100
5.	Butanedioic acid, bis(trimethylsilyl) ester	21.14	247, 172, 147, 73, 45	90	Organic acid derivatives	262	90
6.	d-Mannose, 2,3,4,5,6-pentakis-*O*-(trimethylsilyl)-*O*-methyloxyme (1Z)	28.64	319, 217, 205, 160, 147, 129, 127	97	Carbohydrate	569	95
7.	Ethanedioic acid, bis(trimethylsilyl) ester	17.86	221, 190, 73, 59, 45, 29	83	Organic acid	234	100
8.	Glycine, *N*-(trimethylsilyl)-, trimethylsilyl ester	17.42	204, 176, 147, 102, 73, 45	92	Amino acid derivatives	219	100
9.	l-Isoleucine, *N*-(trimethylsilyl)-, trimethylsilyl ester	20.78	261, 218, 158, 73, 45	91	Amino acid derivatives	275	100
10.	l-Proline, 1-(trimethylsilyl), trimethylsilyl ester	20.84	216, 170, 142, 73, 45	81	Amino acid derivatives	259	100
11.	l-Proline, 5-oxo-1-(trimethylsilyl)-, trimethylsilyl ester	24.15	258, 230, 214, 156, 147, 73, 45	90	Amino acid derivatives	273	100
12.	*N*,*N*-Dimethylglycine, trimethylsilyl ester	12.88	160, 117, 58	94	Amino acid derivatives	175	87.5
13.	Pentanedioic acid, 2-(methoxyimino)-, bis(trimethylsilyl) ester	24.85	304, 288, 198, 147, 73	88	Organic acid derivatives	319	85
14.	Phosphoric acid, bis(trimethylsilyl) 2,3-bis[(trimethylsilyl)oxy] propyl ester	27.06	445, 357, 299, 211, 207, 147, 73, 45	84	Inorganic acid derivatives	460	100
15.	Phosphoric acid, bis(trimethylsilyl) monomethyl ester	18.62	241, 211, 195, 133, 73, 45	91	Inorganic acid derivatives	256	92.5
16.	Propanoic acid, 2-(methoxyimino)-, trimethylsilyl ester	15.50	174, 115, 89, 73, 59, 45	87	Organic acid derivatives	189	90
17.	Propanoic acid. 2-[(trimethylsilyl)oxy]-, trimethylsilyl ester	15.77	219, 191, 147, 117, 73, 45	92	Organic acid derivatives	234	95
18.	Pyrimidine, 2,4-bis[(trimethylsilyl)oxy]-	21.50	255, 241, 147, 99, 59, 45	82	Organic derivatives	256	90
19.	Ribitol 1,2,3,4,5-pentakis-O-(trimethylsilyl)-	26.07	319, 305, 217, 205, 147, 73	88	Carbohydrate	512	100
20.	Serine, bis(trimethylsilyl)-	20.16	219, 188, 159, 144, 132, 116, 103, 88, 75, 73, 45	90	Amino acid derivatives	249	95
21.	Trimethylsilyl 3,4-bis(trimethylsiloxy) cinnamate	31.72	396, 307, 219, 191, 147, 73	84	Organic acid derivatives	396	90
22.	Uridine. 2′,3′,5′-tris-*O*-(trimethylsilyl)-	36.96	370, 299, 259, 217, 169, 147, 73	90	Nucleoside	460	100

### Statistical Analysis

The obtained data set, composed of 69 compounds versus 40 samples, was subjected to statistical analysis. In terms of checking the data distribution, the Shapiro–Wilk test was used. Afterward, depending on the achieved results, the adequate univariate statistical analysis was applied (*t* test or U Mann–Whitney test). The calculations were performed with the use of Statistica (Statistica 10.0, Statsoft, Tulsa, OK, USA).

Along with the univariate statistical analyses, a multivariate one was also carried out, namely principal component analysis (PCA), for checking the data classification and outliers detection, as well as partial least squares discriminant analysis (PLS-DA), also for data classification and prediction. The PCA and PLS-DA models were calculated using Simca Software (Simca P + 12.0, Umetrics, Malmö, Sweden). For the prediction purposes, the data set was divided into calibration set and test set using Kennard–Stone algorithm [15] and duplex algorithm [16]. The PLS-DA model was validated using “leave one out cross validation” (LOOCV) and the model with the both lowest root mean square error of cross validation and number of latent variables was used for the prediction. The validation and sample subset selection was performed in Matlab environment (Matlab 7.0, Mathworks, Natick, MA, USA).

## Results and Discussion

Folk tales, still popular in certain provinces in Poland, carry information about therapeutic properties of numerous substances. In some of them, grasshopper abdominal secretion was notified to be used to stimulate wound healing process. However, there is no scientific evidence till now to rationalize the usage of grasshoppers’ abdominal secretion for medical purposes. Therefore, we decided to analyze the biochemical composition of grasshopper secretion to identify compounds with probable wound healing potential.

It is known, that compounds obtained from natural products can affect several stages of wound healing process. Vitamins (especially A, E and C), alkaloids, terpenoids, polyphenols, tannins, sugars are supposed to support the wound healing process. Their activity is assumed to be due to the modulation of different factors affecting the healing process: skin cells growth, immune cell functions, collagen synthesis, angiogenesis, extracellular matrix, cytokines and growth factors, as well as the oxidant–antioxidant balance of the wound microenvironment [17].

The main aim of the proposed study was to carry out untargeted metabolomic study of water-soluble compounds of grasshoppers’ abdominal secretion. Therefore, due to the lack of knowledge on the composition of water-soluble fraction, we could only suspect what kind of compounds might be present in insect’s secretion. Identification of compounds was based on comparison of the spectra obtained from EI-GC/MS technique with spectra collected in NIST library. Such approach is recommended as one of the most reliable way of compound identification.

The results of this study enable us to present the water-soluble compounds comprised in grasshoppers’ abdominal secretion.

**Fig. 1:**
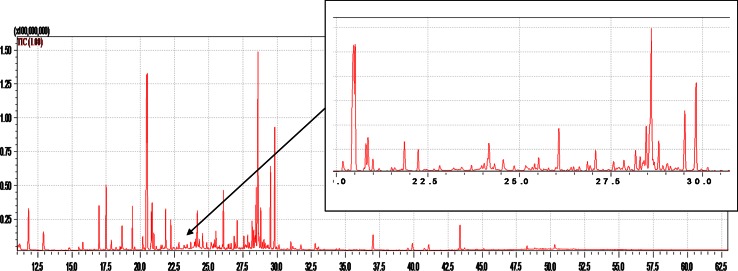
GC/MS chromatogram obtained with the use of scan mode for an extract of grasshoppers’ abdominal secretion; the GC/MS conditions applied are described in “Materials and Methods” section

The obtained profile of the analyzed material contains 2,108 compounds classified into groups of amino acids, organic acids, carbohydrates, alcohols, hydrocarbons, nucleosides and inorganic acids. Exemplary chromatograms of the analyzed material are presented in the Fig. 1.

### Compounds Identified in at Least 80 % of Analyzed Samples

After data preprocessing, we selected 22 compounds identified in all the analyzed samples obtained from *Chorthippus* spp. in at least 80 % in each studied group. The compounds are listed in Table 1.

**Fig. 2:**
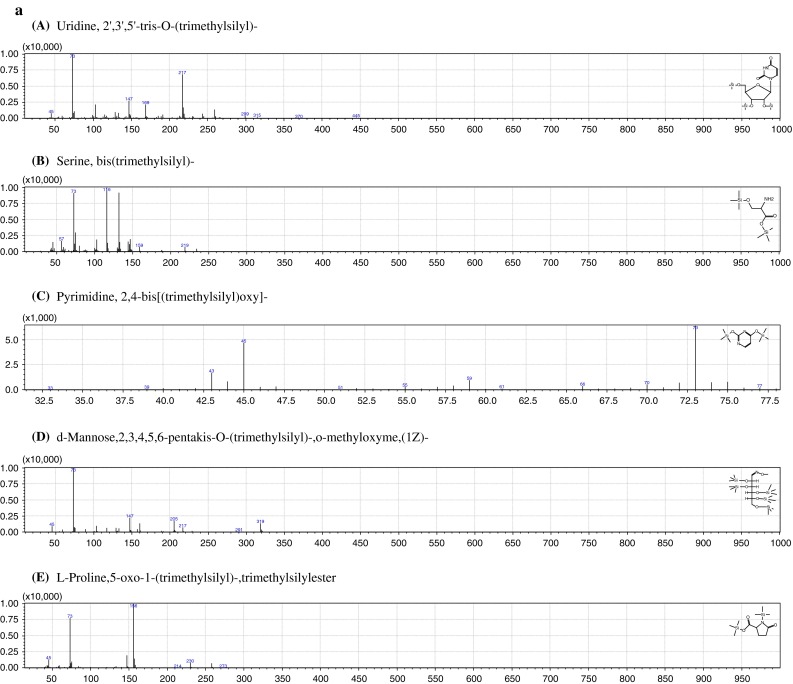

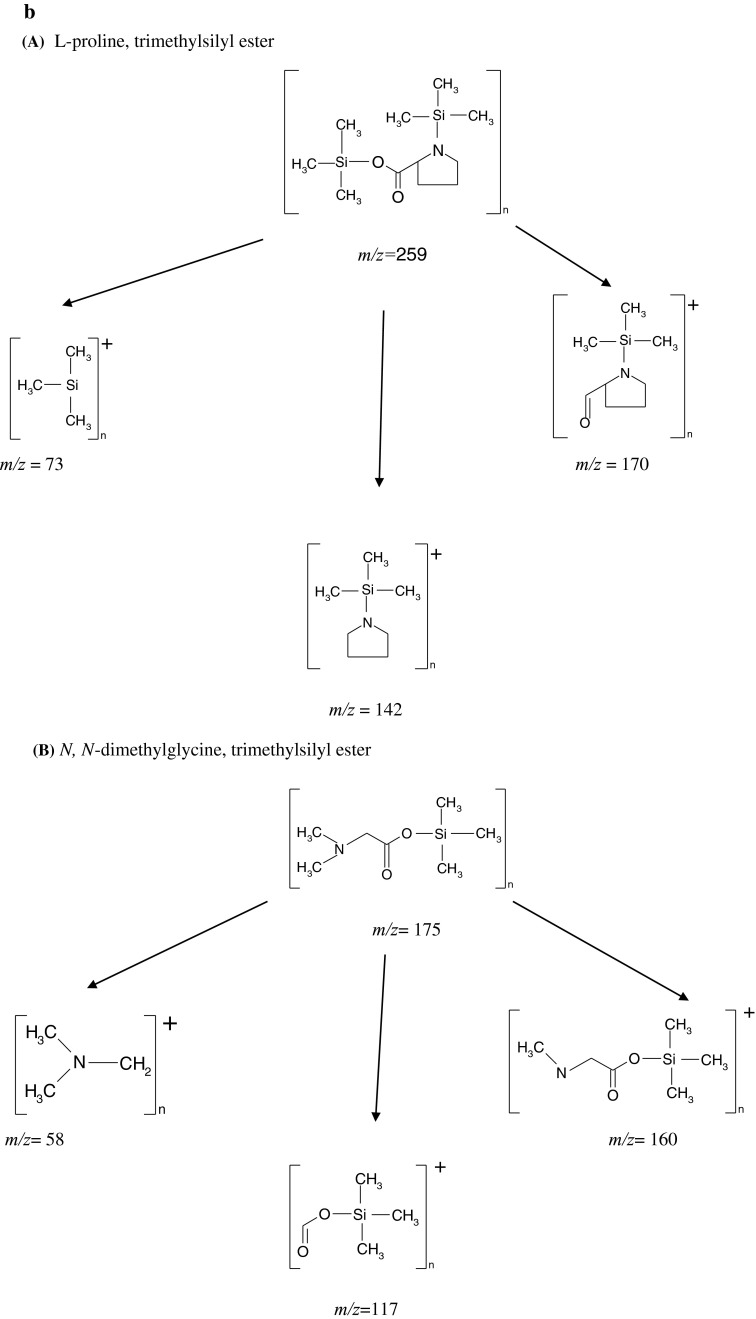
**a** EI mass spectrum for five exemplary analytes present in at least 80 % of all aqueous fraction of grasshoppers’ abdominal secretion: Uridine, A; Serine, B; Pyrimidine, C; Mannose, D and 5-oxo-Proline, E. **b** Proposed fragmentation pathways of two exemplary compounds present in aqueous fraction of grasshopper abdominal secretion at high frequency: **A** proline, trimethylsilyl ester and **B** *N*,*N*-dimethylglycine, trimethylsilyl ester

In these groups we could select: amines, one alcohol, one nucleoside, organic as well as inorganic acids and their derivatives, carbohydrates as well as amino acids and their derivatives. The most abundant group of compounds (9 out of 22 compounds, which constitutes 41 %) was composed of organic acids and their derivatives. The second group of compounds, determined in aqueous fraction of grasshoppers’ abdominal secretion, was composed of amino acids and their derivatives (5 out of 22 compounds, 23 %). Compounds and their features, including characteristic fragmentation patterns, retention times and frequency of appearance in all samples, are presented in Table 1. The mass spectra of five exemplary compounds (Uridine, A; Serine, B; Pyrimidine, C; Mannose, D and 5-oxo-Proline, E), identified in ≥90 % of aqueous fractions extracted from grasshoppers abdominal secretions, are presented in Fig. 2a. Figure 2b presents proposed fragmentation pathways of two exemplary compounds, l-Proline- trimethylsilyl ester (A) and *N,N*-dimethylglycine, trimethylsilyl ester (B).

### Statistical Analysis

The obtained data, after normalization were characterized by normal distribution. Therefore, the *t* test was applied to extract the statistically significant compounds from the two studied groups (Łubianka and Starograd Gdański meadows). As a result, 10 out of 69 compounds have been found to be statistically significant within two groups (*p* < 0.05) and are presented in Table 2.

**Table 2:** Water-soluble compounds recognized as statistically significant for two studied groups of grasshoppers’ abdominal secretion

Compound no.	Compound	*p* value	*t*_R_	Frequency (%)
1.	Phosphonic acid, [2-[(trimethylsilyl)amino]ethyl]-, bis(trimethylsilyl) ester	0.041	22.38	82.5
2.	d-Arabinose, tetrakis(trimethylsilyl)-	0.016	25.78	65
3.	l-Tyrosine, *N*,*O*-bis(trimethylsilyl)-, trimethylsilyl ester	0.003	29.11	60
4.	*n*-Butylamine, *N*,*N*-bis(trimethylsilyl)	0.032	21.57	67.5
5.	Acetic acid, [(trimethylsilyl)oxy]-, trimethylsilyl ester	0.008	16.28	65
6.	Butanedioic acid, bis(trimethylsilyl) ester	0.004	21.14	90
7.	1,4-Butanediamine, *N*,*N*,*N*′,*N′*-tetrakis(trimethylsilyl)-	0.018	26.84	82.5
8.	2-Pyrrolidone-5-carboxylic acid, trimethylsilyl ester	0.019	24.30	87.5
9.	Acetic acid, bis[(trimethylsilyl)oxy]-, trimethylsilyl ester	0.049	23.87	60

**Fig. 3:**
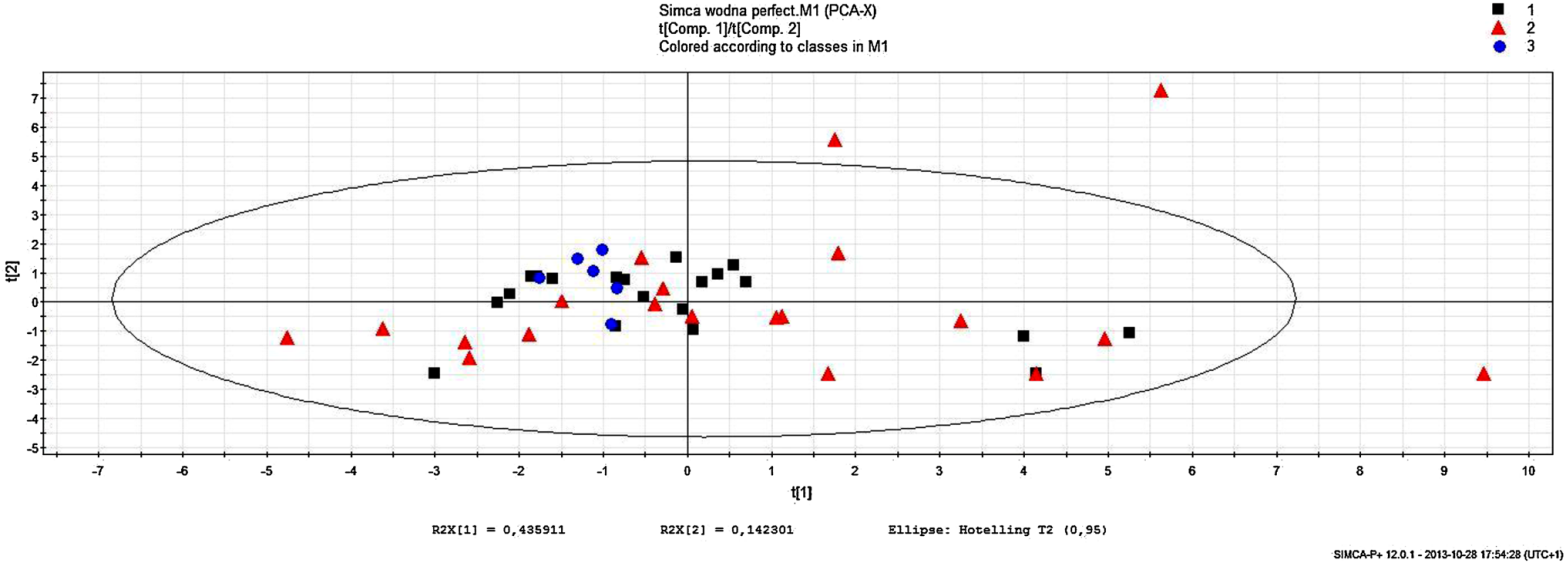
A principal component analysis of GC/MS data. *Red triangles* (1), *black boxes* (2) and *blue dots* (3) correspond to Starogard Gdański insects, insects collected in Łubianka and QC samples, respectively

In multivariate statistical analyses, the principal component analysis (PCA), as an unsupervised chemometric method, and the partial least squares discriminant analysis (PLS-DA), as a supervised one, were applied. PCA analysis was carried out to check general trends in classification of the obtained data as well as to detect outliers. In the proposed manuscript, we present untargeted analysis of water-soluble compounds of grasshopper abdominal secretion. In the untargeted approach, the reliability as well as stability of the method is determined with the use of quality control samples (QCs), which are composed of all the analyzed biological samples mixed together and assayed sequentially in the following manner: at the beginning, to stabilize the equipment, after each eight samples, and at the end of the sequence. Therefore, in analysis of 40 samples, about 5–6 quality control samples were also determined. The confirmation of reliability as well as system stability depends on clustering of QCs among other analyzed samples in a hyperplane determined by the so-called principal components using the principal component analysis. As it can be observed in Fig. 3, the QC samples are classified together in one separate cluster, what proves the analytical stability and reliability of the analytical method during the study. For PCA analysis, that data set after filtration (maximum RSD for QC samples = 40 %) and autoscaling was applied. The results are presented in Fig. 3.

As it is seen in Fig. 3, the separation of each sample group from other clusters has not been evident. Nevertheless, there was a trend in clustering of samples from Łubianka (black boxes), like in the case of samples from Starogard Gdański (red triangles). However, samples from Starogard Gdański were found to be more dispersed. That situation might be a result of species polymorphism within the collected insects. The quality control samples, which indicate the analytical stability of the GC/MS system, were clustered together. It signified that the analytical procedures had been stable and did not have any influence on samples’ classification. The *R* ^2^ and *Q* ^2^ scores, obtained for the proposed models, were calculated as 0.578 and 0.144, respectively. Moreover, as it can be noticed in Fig. 3, some samples could be treated as outliers. To verify that observation, the Hotelling’s test was applied. As a result, two samples have been identified as outliers from Starogard Gdański group (0.99 confidence level) and were excluded from further statistical analysis.

**Fig. 4:**
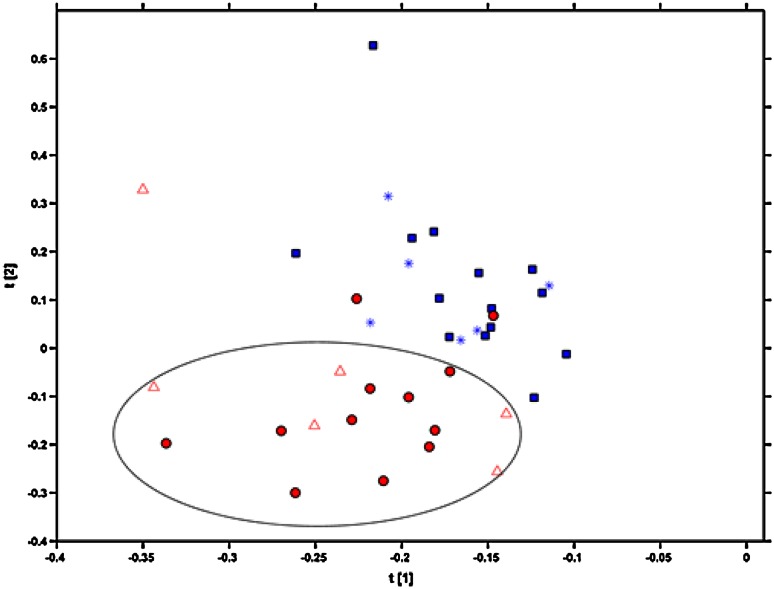
PLS-DA analysis of calibration and test sets. *Red color* corresponds to Starogard Gdański insects, while *blue* one relates to insects collected from Łubianka. The *red dots* and *blue boxes* correspond to samples from training set, while *red triangles* and *blue asterisks* represent samples from test set

To check the prediction ability of the model, the partial least squares discriminant analysis (PLS-DA), was carried out. This method, known as a supervised one, was used for sample classification and prediction. In contrast to PCA analysis, wherein samples are classified according to the data matrix itself, in PLS-DA method the samples’ classification is based on data matrix together with the information about class membership. Before analysis, the data set was scaled and divided into the training set (70 % of samples) and test set (30 % of samples), using Kennard–Stone and duplex algorithm, which selects samples based on Euclidean distance and mean value, respectively. The both training sets were validated using leave one out cross validation. Taking into consideration the lowest root mean square error of cross validation and the number of latent variables after validation, the Kennard–Stone algorithm was chosen. Figure 4 presents the PLS-DA classification of training set and test set from both the studied groups (Łubianka and Starogard Gdański meadows).

As it can be noticed in Fig. 4, two samples from training set (red dots) and one sample from test set (red triangle), both derived from Starogard Gdański group, were wrongly classified. The remaining samples were clustered together (the area in the ellipse). Concerning samples from Łubianka, only one sample from training set (blue box) was classified to a wrong group. All samples from the test set derived from Łubianka (blue asterisks) were correctly classified and were clustered into one group. The samples’ classification presented in Fig. 4 was in agreement with the calculations performed with the PLS-DA model, that were presented in Table 3. The correct classification rates for the training set and the test set were 88 and 91 %, respectively. Based on the PLS-DA model, created using the training set, all the samples from Łubianka and 5 out of 6 samples from Starogard Gdański were predicted correctly.

**Table 3:** Calculations of PLS-DA model’s parameters

Parameter	PLS-DA LV (*n* = 2)
Correct classification rate for training set	Overall: 88 %
Łubianka samples (*n* = 14)	93 %
Starogard Gdański samples (*n* = 12)	83 %
Correct classification rate for test set	Overall: 91 %
Łubianka samples (*n* = 6)	100 %
Starogard Gdański samples (*n* = 6)	83 %

Correct classification rate denotes the percentage of correctly classified samples in the calibration and test set

*LV* latent variables

We can assume that the analysis using a larger number of samples may reveal even better classification and prediction results. Therefore, in the future, two or three times larger sample subsets will be determined and statistically analyzed.

### The Most Abundant Compounds Identified

In the presented study 22 compounds with frequency level ≥80 % were identified. Among them one amine, one alcohol, one nucleoside, one organic derivatives, two inorganic acids and their derivatives, seven organic acids and their derivatives, two carbohydrates and seven amino acids with their derivatives were detected. We focused not only on the statistically significant compounds differentiating the samples collected from Starogard Gdański and from Łubianka meadows, but in the description below we also indicate the compounds identified in samples from both groups. Our goal was to assess potential of the grasshopper abdominal secretion in wound healing process. Especially, carbohydrates and amino acids are necessary for proper tissue regeneration, not only due to their supposed wound healing potential but also due to their nourishing properties.

### Amine

Among 22 compounds, one amine with frequency higher than 80 % of the analyzed samples, was identified, namely 1,4 butanediamine. 1,4 Butanediamine is known as putrescine, natural endogenous polyamine. According to the literature, putrescine is present in each living cell [18]. Its biological activity is important in numerous metabolic pathways and physiological processes, like blood pressure regulation, body temperature stability, stomach pH as well as brain activity. On the other hand, it can be toxic if a certain concentration is exceeded [19].

### Nucleoside

As a second compound determined in all the samples (100 %) of the studied data set, was a pyrimidine nucleoside—uridine. Uridine is a small molecule, essential for RNA structure. Its levels can differ in normal and pathological states of the organism [20]. Uridine plays a crucial role in cell pathways and has been associated with glycogen production, as well as with glucose metabolism. Uridine is one of modulators of sugars metabolism in each cell. Moreover, it is well known that the injured tissue requires increasing amounts of glucose and an increased activity of the pentose cycle [21].

### Amino Acids and Their Derivatives

According to the literature, amino acids play an important role in wound healing [22–25]. In the obtained data set, seven amino acids and their derivatives (phenylalanine, glycine, isoleucine, proline, 5-oxo-proline, *N*,*N*-dimethylglycine, serine) were present at the 80 % level of frequency.

One of the most prevalent amino acid in the tested samples was glycine (present with 100 % frequency). Glycine, as well as its derivatives, like dimethylglycine, can be a final product of choline metabolism but also could be formed from other amino acids, like threonine and serine. In mammalian organism, glycine and its derivatives are known as compounds with detoxifying properties in wound healing process. Moreover, it plays a significant role in hemoglobin and collagen synthesis. The latter one is a highly important protein in wound healing as it is one of the main constituents of the connective tissue. Glycine and proline, amino acids found in grasshopper abdominal secretions, are structural components of collagen. Therefore, deficiency of either glycine or proline may result in impaired wound healing [22].

Another amino acid—l-proline, stimulates the collagen production, as was mentioned previously. In addition, it is responsible for a youthful skin look [23].

Serine, identified in all the samples with high frequency, is another amino acid with important biological activity. It takes part in tryptophan and creatine formation. As a part of phospholipids, it is also involved in membrane and immunoglobulin’s formation and is a structural component of myelin and neuron sheath [24, 25].

### Organic Acids and Their Derivatives

2-Pyrrolidone-5-carboxylic acid is also known as a pyroglutamic acid. In the gamma-glutamyl cycle, this small molecule is an intermediate responsible for amino acids transfer into cells [26].

Systematic name of butanedioic acid, present in the analyzed samples with 90 % frequency, is succinic acid. Succinic acid is water-soluble compound, naturally present in plant and animal tissues. Besides uridine, succinic acid plays a crucial role in the Krebs cycle [27].

Another compound determined in aqueous fraction of grasshoppers’ abdominal secretion is ethanedioic acid, known also as oxalic acid. Oxalic acid is found in plants, vegetables, animals and fungi [28]. The concentration of oxalic acid in organisms depends on age, weather conditions and tissue. The recent study shows that the final product of oxalic acid metabolism is H_2_O_2_. The production of hydrogen peroxide causes an increase in number of phagocytes, which are responsible for breaking down different pathogens [28]. Çalişkan [29] reported that the concentration of oxalic acid is higher in leaves infected by fungus *Sclerotinia sclerotiorum*.

Propionic acid is one of the smallest acids occurring naturally. It is produced by several species of anaerobic bacteria [30]. Natural propionic acid is a product of metabolism of several carbon sources (glucose, maltose, xylose, sucrose, lactate, glycerol), produced mainly by *Propionibacterium*. This naturally occurring acid can inhibit cell growth due to its ability to cross the cell membrane and change the pH value of the cytosol, which results in growth inhibition of some bacteria, fungi and yeasts [30].

Trimethylsilyl 3,4-bis(trimethylsiloxy) cinnamate is a product of derivatization of cinnamic acid. Cinnamic acid, present in 90 % of the samples, naturally occurs in cinnamon oil or shea butter, however, it is present in all plants, but at small concentrations. Cinnamic acid is known from its ability to exert anti-tumor, cytoprotective and antioxidant effects on endothelial cells [31].

### Carbohydrates

Ribitol, a carbohydrate found in all the samples, known also as adonitol, can be formed as a product of ribose reduction, which naturally occurs in plants, e.g., *Adonis vernalis*. This polyol is used as a cryoprotective agent and can increase cold tolerance in insects [32]. Storey and Storey [33] presented the metabolism of glycerol pathway, where polyols, known as compounds with cryo–protective activities, are metabolized. Storey and Storey found that ribitol acting as a cryoprotectant is responsible for stabilizing the protein structure, in which it increases membrane integrity. Moreover, in freezing tissues, it is also a significant factor for reducing osmotic fluctuations [33].

Another compound determined in grasshopper abdominal secretion is mannose. Mannose was found in *Aulocara elliotti* eggs by Quickenden [34]. It was observed that presence and concentration of mannose is strictly dependent on insect growth stage. The presence of mannose was confirmed in early pre-diapause eggs. In diapause and post-diapause eggs this hydrocarbon was not detected [34].

## Conclusion

In the current study, an innovative method for qualitative determination of compounds from aqueous fraction of grasshopper *Chorthippus* spp. abdominal secretion was proposed. The sample preparation relied on Bligh and Dyer extraction, while separation and identification of compounds was based on gas chromatography coupled with mass spectrometry technique (GC/MS/MS). In the samples, a variety of chemical groups of compounds were identified, mainly as amino acids, carbohydrates, amines, nucleosides, organic and inorganic acids. Several of them were recognized as possessing antimicrobial, antifungal and antioxidant activities, which might be beneficial in wound healing process. The presumed wound healing potential of aqueous extract from grasshopper abdominal secretion needs confirmation in studies employing in vivo and/or in vitro tests.
